# New aspects in fenestrated capillary and tissue dynamics in the sensory circumventricular organs of adult brains

**DOI:** 10.3389/fnins.2015.00390

**Published:** 2015-10-27

**Authors:** Seiji Miyata

**Affiliations:** Department of Applied Biology, Kyoto Institute of TechnologyKyoto, Japan

**Keywords:** neural stem cells (NSCs), angiogenesis, TRPV1, TLR4, homeostasis, inflammation, VEGF, blood-brain barrier (BBB)

## Abstract

The blood–brain barrier (BBB) generally consists of endothelial tight junction barriers that prevent the free entry of blood-derived substances, thereby maintaining the extracellular environment of the brain. However, the circumventricular organs (CVOs), which are located along the midlines of the brain ventricles, lack these endothelial barriers and have fenestrated capillaries; therefore, they have a number of essential functions, including the transduction of information between the blood circulation and brain. Previous studies have demonstrated the extensive contribution of the CVOs to body fluid and thermal homeostasis, energy balance, the chemoreception of blood-derived substances, and neuroinflammation. In this review, recent advances have been discussed in fenestrated capillary characterization and dynamic tissue reconstruction accompanied by angiogenesis and neurogliogenesis in the sensory CVOs of adult brains. The sensory CVOs, including the organum vasculosum of the lamina terminalis (OVLT), subfornical organ (SFO), and area postrema (AP), have size-selective and heterogeneous vascular permeabilities. Astrocyte-/tanycyte-like neural stem cells (NSCs) sense blood- and cerebrospinal fluid-derived information through the transient receptor potential vanilloid 1, a mechanical/osmotic receptor, Toll-like receptor 4, a lipopolysaccharide receptor, and Nax, a Na-sensing Na channel. They also express tight junction proteins and densely and tightly surround mature neurons to protect them from blood-derived neurotoxic substances, indicating that the NSCs of the CVOs perform BBB functions while maintaining the capacity to differentiate into new neurons and glial cells. In addition to neurogliogenesis, the density of fenestrated capillaries is regulated by angiogenesis, which is accompanied by the active proliferation and sprouting of endothelial cells. Vascular endothelial growth factor (VEGF) signaling may be involved in angiogenesis and neurogliogenesis, both of which affect vascular permeability. Thus, recent findings advocate novel concepts for the CVOs, which have the dynamic features of vascular and parenchymal tissues.

## Introduction

**Figure 1:**
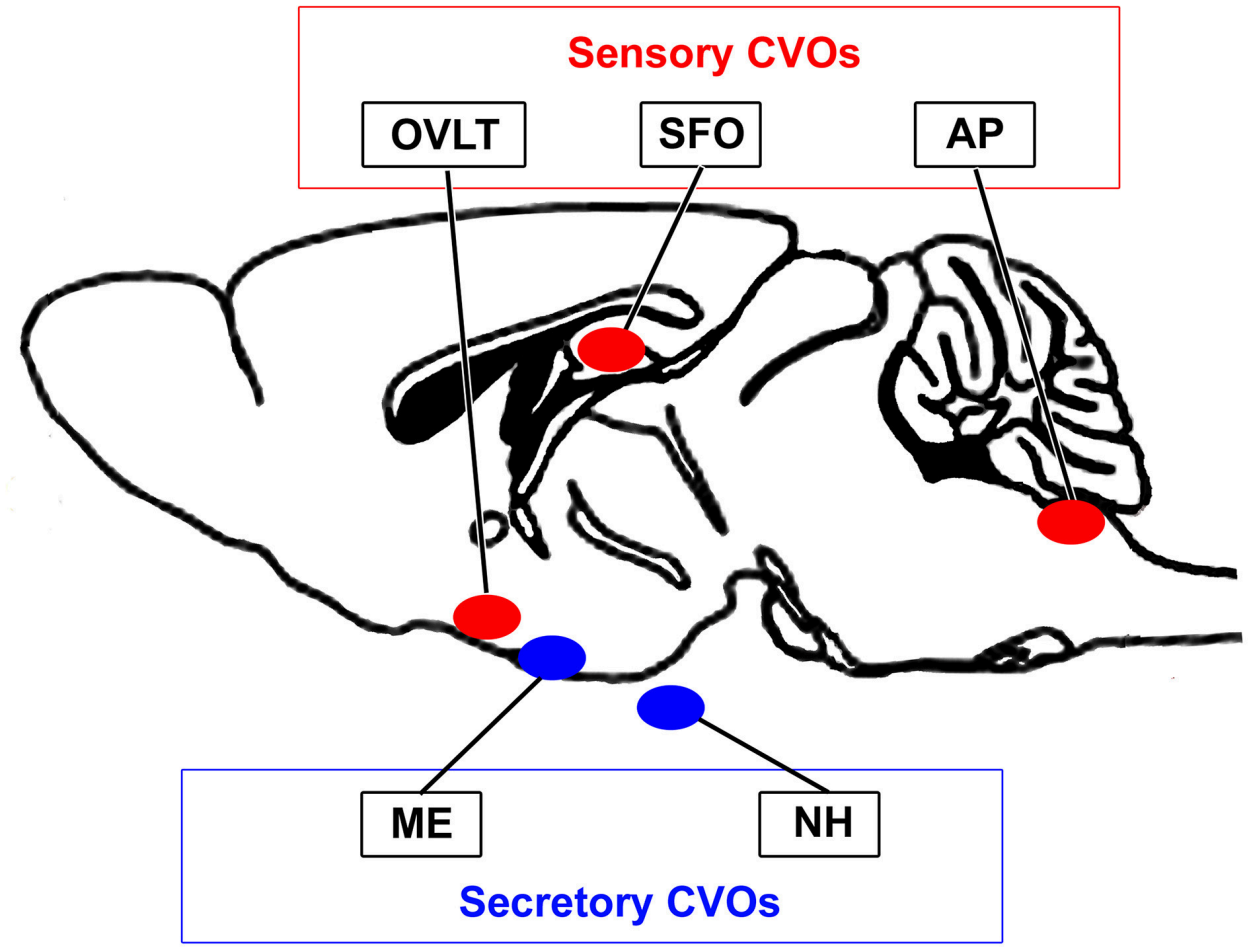
**Schematic illustration showing localization of the sensory and secretory CVOs in adult rodent brains**.

The blood-brain barrier (BBB) is generally composed of endothelial tight junctions and maintains the chemical composition of the neuronal environment for the proper functioning of neuronal circuits by preventing the entry of blood-derived substances in adult brains. Therefore, dysfunctions in the BBB result in the diffusion of blood-derived substances into the brain parenchyma and subsequent neuronal damage (Zlokovic, 2011). In 1958, the brain regions located around brain ventricles were accordingly named “circumventricular organs (CVOs)” (Hofer, 1958). The CVOs were subsequently referred to as the “windows of the brain” because they have distinct features, such as fenestrated capillaries, relatively large perivascular spaces, and highly specialized ependymal cells (Weindl, 1973; Gross and Weindl, 1987). Three sensory and four secretory CVOs have been identified to date (Leonhardt, 1980; Cottrell and Ferguson, 2004). The sensory CVOs, including the subfornical organ (SFO), organum vasculosum of the lamina terminalis (OVLT), and area postrema (AP), permit brain cells to monitor blood- and cerebrospinal fluid (CSF)-derived information, which is then transmitted to other brain regions (Johnson and Gross, 1993; Sisó et al., 2010a,b). The secretory CVOs, the so-called neurosecretory regions consisting of the neurohypophysis (NH), median eminence (ME), and pineal gland, release large amounts of brain-derived hormones into the blood circulation from brain neurons (Miyata et al., 2001; Miyata and Hatton, 2002; Ciofi et al., 2009). In addition to these regions, the choroid plexus, which is present in most ventricular systems and produces cerebrospinal fluid (CSF), is regarded as a CVO because it has fenestrated capillaries, but lacks neurons. The subcommissural organ has also been proposed as a CVO, but does not possess fenestrated capillaries (Petrov et al., 1994). Therefore, the fenestrated capillaries of the CVOs permit communication between the brain parenchyma and blood (for a review, see Johnson and Gross, 1993; Rodríguez et al., 2010; Sisó et al., 2010a,b; Sladek and Johnson, 2013; Noda and Hiyama, 2015; Figure 1).

The sensory CVOs play important roles in body fluid homeostasis by sensing plasma Na^+^ levels and osmotic pressure (Sladek and Johnson, 2013; Noda and Hiyama, 2015). SFO neurons have been shown to respond to increases in angiotensin II and Na^+^ levels in plasma and the CSF (Fitzsimons, 1975; Ishibashi et al., 1985; Tiruneh et al., 2013). These CVOs detect circulating hormones such as cholecystokinin, amylin, and ghrelin (McKinley et al., 2003; Fry and Ferguson, 2009). Disruptions to the sensory CVOs were found to markedly attenuate thermal tolerance, such as attenuated salivation, and also impair cardiovascular responses to heat stress (Johnson and Gross, 1993; Whyte and Johnson, 2005; Sladek and Johnson, 2013). A previous study reported that emetic chemicals stimulated chemosensitive receptors in the AP and solitary nucleus in order to induce vomiting and nausea (Hornby, 2001). Toll-like receptor 4 (TLR4) mRNA was previously reported to be strongly expressed in the sensory CVOs of adult mice (Laflamme and Rivest, 2001; Chakravarty and Herkenham, 2005; Nakano et al., 2015). Furthermore, the peripheral administration of lipopolysaccharide (LPS) activated the signal transducer and activator of transcription factor 3 (STAT3) in the sensory CVOs (Harré et al., 2002, 2003; Rummel et al., 2005; Nakano et al., 2015).

In the secretory CVOs, oxytocin (OXT), and arginine-vasopressin (AVP) are secreted into the blood circulation from axonal terminals in the NH (Miyata and Hatton, 2002) and adenohypophyseal hormone-releasing factors are secreted from hypothalamic axonal terminals in the ME (Müller et al., 1999; Prevot et al., 2007). The axonal terminals of hypothalamic neurons have been shown to exhibit neurovascular and neuroglial structural plasticity in the ME during the estrous cycle (Prevot, 2002; Ojeda et al., 2008) and the NH during dehydration and lactation (Miyata et al., 2001; Miyata and Hatton, 2002). In the NH, Notch signaling has been associated with neurovascular and neuroglial plasticity (Miyata et al., 2004, 2005; Mannari and Miyata, 2014).

Thus, the CVOs are specialized brain regions that permit the direct sensing of blood information and secretion of hypothalamic neuropeptides via their fenestrated capillaries. Accumulating evidence has demonstrated the crucial roles of the CVOs in many physiological regulatory pathways, such as the homeostasis of osmolarity, Na^+^ levels, body temperature, energy balance, the chemoreception of blood-derived substances, and neuroinflammatory responses. Moreover, the CVOs have been implicated in several diseases, such as sepsis, stress, trypanosomiasis, autoimmune encephalitis, systemic amyloidosis, and prion infections (for review, see Sisó et al., 2010a,b), suggesting that more attention needs to be paid to these organs. In this review, recent advances in the CVOs have been discussed, with a focus on the sensory CVOs. The fenestrated capillaries of these regions have size-selective and low permeabilities, which are markedly different from those of peripheral tissues. Furthermore, fenestrated capillaries undergo continuous angiogenesis and reconstruct their architecture and density depending on the signaling of vascular endothelial growth factor (VEGF), which largely affects blood-brain communication. Tanycyte- and/or astrocyte-like neural stem cells (NSCs) are present in the sensory CVOs, in which they produce new neurons and glial cells and respond to blood- and CSF-derived information by sensing proteins and surrounding mature neurons as a barrier to protect them from blood- and CSF-derived toxic substances.

## Size-selective vascular permeability

**Figure 2:**
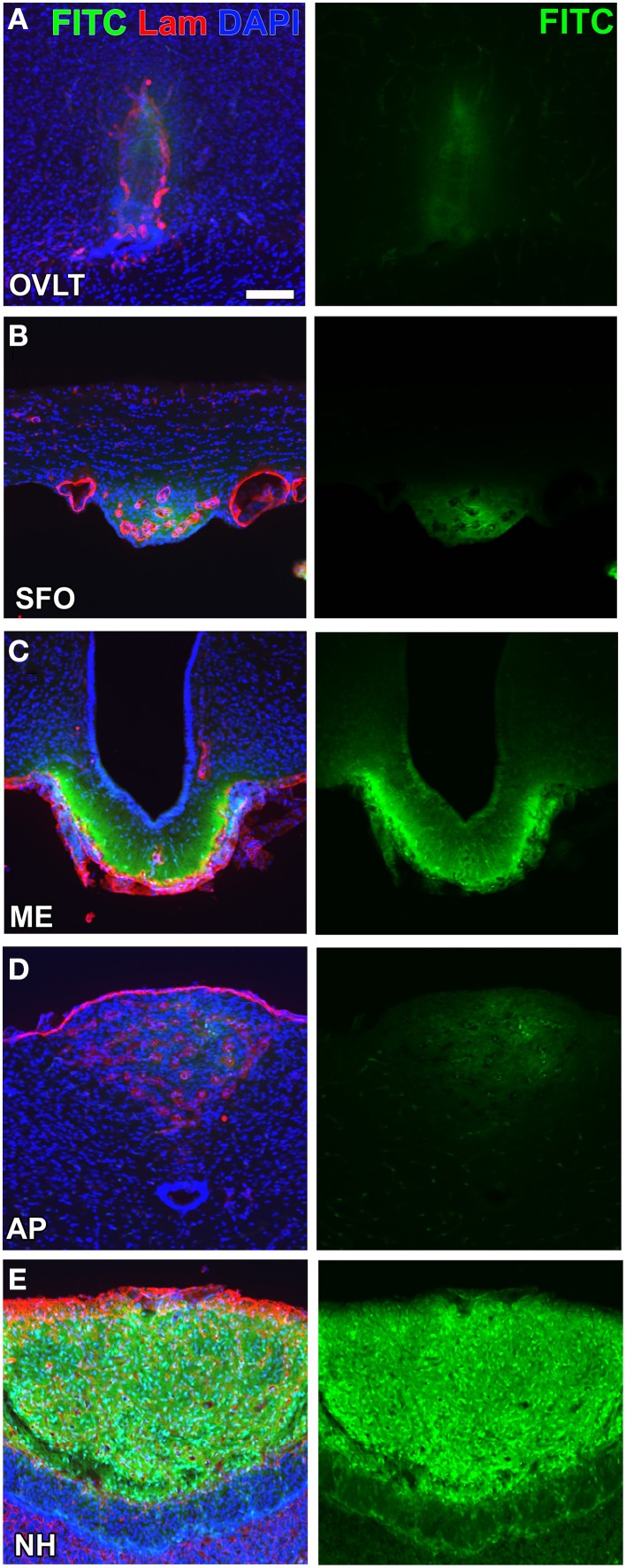
**Different vascular permeabilities between the sensory and secretory CVOs**. The extravascular fluorescence of the LMW fluorescent tracer FITC was stronger in the secretory CVOs **(C,E)** than in sensory CVOs **(A,B,D)**. Scale bar = 50 μm. Lam, laminin; DAPI, 4',6-diamidino-2-phenylindole. Confocal micrographs are rearranged with permission from Springer-Verlag (Morita and Miyata, 2012).

A consensus has not yet been reached regarding the definition of and methods for examining vascular permeability (Nagy et al., 2008). Most fluorescent tracer substances are taken up by brain endothelial cells (Antohe et al., 1997; Miyata and Morita, 2011; Gonnord et al., 2012) and, thus, the immunohistochemical staining of capillaries is essential for accurately determining vascular permeability (Daneman et al., 2010). Moreover, the most commonly used low-molecular-weight (LMW; MW < 10,000) fluorescent tracers, fluorescein (*MW* = 332; Hawkins and Egleton, 2008) and Evans blue (*MW* = 961; Del Valle et al., 2008), dislocate and diffuse during/after saline wash, fixation, and storage procedures. A more reliable method was recently developed to examine the vascular permeability of LMW substances by employing fluorescein isothiocyanate (FITC; *MM* = 390, Miyata and Morita, 2011). FITC binds covalently to the primary amine groups of cellular components in order to form a stable thiourea link and is considered useful for immunohistochemistry without diffusion or dislocation (Miyata and Morita, 2011). The vascular permeability of FITC was previously shown to be markedly higher in the secretory CVOs than in the sensory CVOs (Morita and Miyata, 2012; Figure 2). Lysine-fixable dextran 3k (*MW* = 3000) also showed high vascular permeability in the sensory CVOs, whereas dextran 10k (*MW* = 10000) was impermeable (Willis et al., 2007; Morita et al., 2015a), indicating that the MW size cut-off is less than 10,000. In the ME, the permeability of dextran tracers was shown to decrease between MW 20,000 and 40,000 using *in vivo* multiphoton microscopy (Schaeffer et al., 2013). The high vascular permeability of LMW substances in the secretory CVOs has been attributed to the secretion of large amounts of hypothalamic neuropeptides into the blood circulation.

The high-molecular-weight (HMW) tracers, bovine serum albumin (*MW* = 70,000), dextran 10k (*MW* = 10,000), and dextran 70k (*MW* = 70,000), are permeable to the endothelial cell layer and inner basement membrane in the CVOs. However, the extravasation of these HMW (MW ≧ 10,000) tracers was previously shown to be markedly lower in the CVOs than in peripheral tissues (Faraci et al., 1989; Willis et al., 2007; Morita et al., 2013b, 2015a). These findings differed from those of other studies in which a large amount of horseradish peroxidase (*MW* = 40,000) diffused into the parenchyma in the OVLT (Herde et al., 2011) and ME (Broadwell et al., 1983; Rodríguez et al., 2010). Horseradish peroxidase is known to be incorporated by mannose receptor-mediated transcellular and clathrin-mediated transcytosis routes (Ellinger and Fuchs, 2010). In the medial basal hypothalamus, wheat germ agglutinin lectin (*MW* = 38,000) was shown to be taken up by tanycytes in the arcuate nucleus (Arc) via cellular internalization (Peruzzo et al., 2004; Morita et al., 2013a). Although, HMW substances are often incorporated by cellular internalization, the passive diffusion of substances through fenestrations is dependent on size and charge (Ballerman and Stan, 2007). Thus, necessary bioactive HMW substances are incorporated by cellular internalization, although the vascular permeability of HMW substances is essentially limited or low in the CVOs.

## Extraendothelial barriers to protect neurons

The endothelial tight junction maintains a constant chemical environment for the proper functioning of brain neuronal circuits in most brain regions by inhibiting the entry of blood-derived substances and ions (Zlokovic, 2011). For example, the plasma level of the excitatory neurotransmitter glutamate is 500–1000 μM, whereas its extracellular brain level is only 0.2–2 μM (Hawkins, 2009). Blood-derived HMW proteins, immunoglobulins, albumin, plasmin, and fibrin have been shown to induce neuronal and neurovascular damage (Zlokovic, 2011). However, neural and vascular damage has not been reported in the sensory CVOs in spite of the presence of fenestrated capillaries, suggesting the occurrence of extraendothelial barriers.

**Figure 3:**
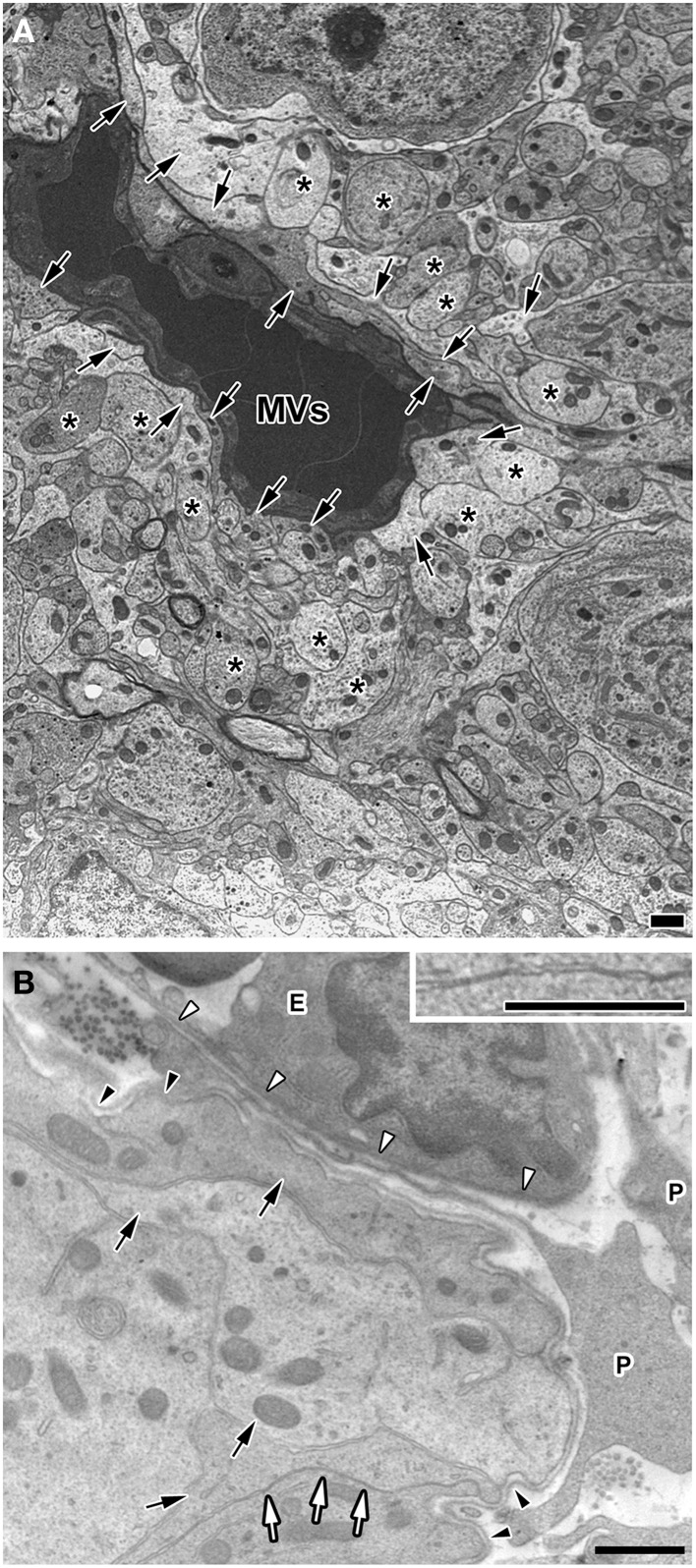
**Electron micrographs showing coverage of fenestrated capillaries by cellular processes of astrocyte-/tanycyte-like NSCs and dendrites and a wide perivascular space in the adult mouse**. The fenestrated capillaries of the AP were surrounded by the cellular processes of astrocyte-/tanycyte-like NSCs and dendrites **(A)**. There was a wide perivascular space including pericytes between the inner and outer basement membranes of the OVLT. Cellular processes of astrocyte-/tanycyte-like NSCs were often found to make contact with the outer basement membrane **(B)**. Solid arrows and asterisks show the cellular processes of astrocyte-/tanycyte-like NSCs and dendrites, respectively. Open arrows and inset revealed that cellular membranes were tightly juxtaposed with each other. Open and solid arrowheads indicate the inner and outer basement membranes, respectively. E, endothelial cell; P, pericyte. Scale bars = 1 μm. Electron micrographs are rearranged with permission from Springer-Verlag (Morita et al., 2015a).

Electron microscopic studies have demonstrated the presence of trans-endothelial pores 30–80 nm in diameter in the sensory CVOs (Delmann, 1987; Dellmann, 1998; Willis et al., 2007) and the ME (Monroe and Holmes, 1983). The endothelial cells of the sensory CVOs have been shown to express plasmalemma vesicle protein-1 (PV-1), which is an integral membrane protein associated with trans-endothelial pores (Ciofi et al., 2009). Most blood-derived HMW substances are permeable to the monolayer of endothelial cells and the inner basement membrane, but impermeable to the outer basement membrane in the sensory CVOs, which results in their accumulation between the inner and outer basement membranes (Faraci et al., 1989; Willis et al., 2007; Morita and Miyata, 2012; Morita et al., 2015a). Although, requisite blood-derived HMW substances are permeable to the outer basement membrane and reach parenchyma cells, the mechanisms by which they pass through the outer basement membrane have not yet been elucidated in detail. The CVOs consist of relatively large perivascular spaces containing pericytes, fibroblasts, and a few microglia between the inner and outer basement membranes (Faraci et al., 1989; Dellmann, 1998; Morita and Miyata, 2012; Figure 3). The basement membrane component, laminin, has been shown to function as a barrier by impeding the movement of large and charged molecules (Hallmann et al., 2005). Laminin is a trimeric molecule comprised of α-, β-, and γ-subunits, and endothelial cells generate laminins-411 (α4β1γ1) and -511 (α5β1γ1) in any capillary, whereas astrocytes produce laminins-111 (α1β1γ1) and -211 (α2β1γ1), specifically in the brain (Hallmann et al., 2005). The expression of laminin-111 was previously reported to be stronger at the outer basement membrane than at the inner basement membrane in the sensory CVOs (Morita et al., 2013b, 2015a) and the NH (Furube et al., 2014). The lack of laminin β2 in the kidney glomerular basement membrane was found to markedly elevate ferritin (*MW* = 450,000) permeability (Jarad et al., 2006), indicating that the glomerular basement membrane serves as a barrier to HMW substances (Suh and Miner, 2013). Thus, the outer basement membrane may act as a size-selective filter to blood-derived HMW substances in the sensory CVOs.

**Figure 4:**
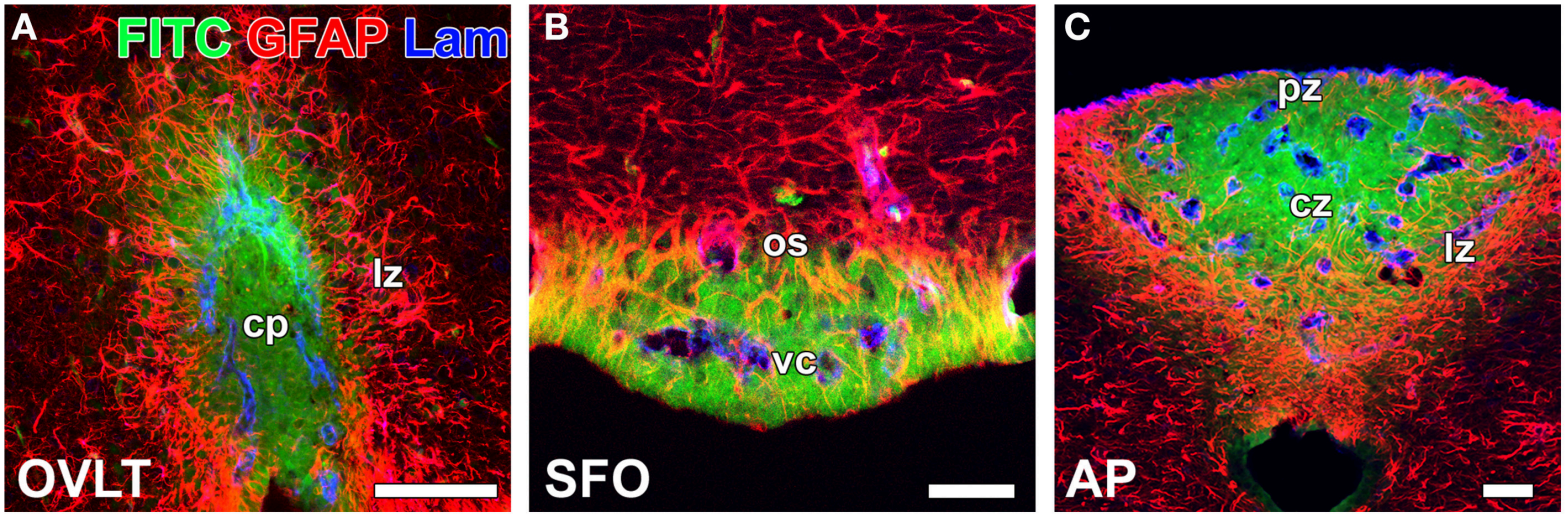
**Heterogeneous vascular permeability of the LMW fluorescent tracer FITC and diffusion barrier of GFAP-positive NSCs in the sensory CVOs of adult mice**. The fluorescent intensity of blood-derived FITC was stronger at the central part of the OVLT **(A)**, SFO **(B)**, and AP **(C)** than at the distal part. The cellular processes of GFAP-positive NSCs were very dense at the distal part of each CVO, and FITC did not diffuse to the outside of the sensory CVOs beyond GFAP-positive NSCs. Scale bars = 50 μm. cp, capillary plexus; cz, central zone; lz, lateral zone; os, outer shell; pz, periventricular zone; vc, ventromedial core. Photomicrographs are rearranged with permission from Springer-Verlag (Morita et al., 2015a).

In contrast to HMW substances, blood-derived LMW substances are permeable to the outer basement membrane and easily reach parenchyma cells as described above. However, the central parts of capillaries in the sensory CVOs were found to exhibit higher vascular permeability to LMW substances than the distal parts (Morita et al., 2015a; Figure 4 and Table 1). Tight junctions are a physical barrier in the BBB between endothelial cells and the blood circulation that prevent the free movement of substances and protect neurons from toxic substances (Saunders et al., 2008). In the sensory CVOs, the expression of claudin-5, occludin, and zonula occludens-1 (ZO-1) was not detected in the OVLT, SFO (Langlet et al., 2013b), or AP (Willis et al., 2007; Norsted et al., 2008; Langlet et al., 2013b), whereas that of ZO-1 was observed in a subpopulation of capillaries in the sensory CVOs (Petrov et al., 1994). A recent study reported that the vascular expression of occludin, claudin-5, and ZO-1 was absent in the central parts of the sensory CVOs, but was present in the distal parts (Morita et al., 2015a). This heterogeneous distribution pattern of tight junction proteins is consistent with the vascular permeability of LWM substances. On the other hand, a heterogeneous distribution pattern for PV-1 was not observed in the sensory CVOs (Ciofi et al., 2009; Morita et al., 2015a). These findings suggest that LMW substances pass through paracellular avenues through inter-endothelial cell junctions rather than PV-1-positive trans-endothelial pores (Table 1).

**Table 1:** **Heterogeneity of endothelial protein expression and vascular permeability in the sensory CVOs of the adult mouse, as summarized from Morita et al. (2015a)**.

	**OVLT**		**SFO**		**AP**		
	**Cp**	**lz**	**os**	**vc**	**pz**	**cz**	**Iz**
Claudin-5	-	+++	+++	-	-	-	+
Occludin	-	-	-	-	-	-	+
ZO-1	-	++	-	-	-	-	+
VE-cadherin	+++	+++	+++	+++	+++	+++	+++
PV-1	+++	+++	+++	+++	+++	+++	+++
FITC 30 s	+	-	-	+	-	+	-
FITC 5 min	+++	+	+	+++	+	+++	+

*Refer to photographs in Figure 4 for more information on subdivisions and permeability; cp, capillary plexus; cz, central zone; lz, lateral zone; os, outer shell; pz, periventricular zone; vc, ventromedial core*.

+*++, strong; ++, moderate; + weak; -, not detected*.

In the secretory CVOs and the ME, _1_ tanycytes extend their cellular processes along the border between the ME and Arc and form adherent and tight junctions between tanycyte processes and between tanycytes and neurosecretory axons (Peruzzo et al., 2000; Mullier et al., 2010). The tanycyte border acts as a barrier separating the ME and Arc (Rodríguez et al., 2010). However, information regarding how neurons are protected from blood-derived neurotoxic and/or bioactive substances in the sensory CVOs is limited. The sensory CVOs possess a large number of neurons and complex neuronal connections in order to integrate blood-derived information and send it to other brain regions (Johnson and Gross, 1993; Sisó et al., 2010a,b). In the sensory CVOs, the density of glial fibrillary acidic protein (GFAP)-positive astrocyte-/tanycyte-like cells appears to be higher in the distal parts than in the central parts of the sensory CVOs (Figure 4). A recent study identified these GFAP-positive astrocyte-/tanycyte-like cells as NSCs (Furube et al., 2015). The diffuse and punctate immunoreactivity of ZO-1 was detected at the parenchyma in the AP (Wang et al., 2008; Maolood and Meister, 2009). The punctuate immunoreactivities of occludin and ZO-1 have been associated with astrocyte-/tanycyte-like NSCs in the sensory CVOs (Morita et al., 2015a). Using conventional and freeze-fracture electron microscopy, tight junctions, arranged in several parallel and helical rows, were observed at the cellular processes of astrocyte-/tanycyte-like NSCs in the sensory CVOs (Krisch et al., 1978). The expression of ZO-1 and occludin was previously shown to be induced in astrocytes in peri-infarct areas during the recovery processes following stroke (Yang et al., 2007, 2013). Numerous layers of the cellular processes of astrocyte-/tanycyte-like NSCs have been found to surround the outer basement membrane of capillaries and neuronal somata in the sensory CVOs (Watanabe et al., 2006; Morita et al., 2015a; Figure 3). Tracer experiments revealed that the blood-derived LMW tracer Dex3k and FITC did not diffuse to the outside of the sensory CVOs beyond astrocyte-/tanycyte-like NSCs (Figure 4). Thus, in the sensory CVOs, tight junctions between astrocyte-/tanycyte-like NSCs and their coverage of fenestrated capillaries and neural somata are possible extraendothelial barriers that protect neuronal circuits from blood-derived neurotoxic and bioactive substances. Moreover, the tanycytes of the sensory CVOs were shown to possess long processes that project into the fenestrated capillary network and display well-organized tight junctions around their cell bodies, indicating that tanycytes act as CSF-brain barriers (Langlet et al., 2013b; for a review, see Rodríguez et al., 2010).

In addition to physical barriers, the CVOs possess a protective mechanism for blood-derived neurotoxic substances. Microglia in the sensory CVOs were found to proliferate robustly in response to a single peripheral inflammation stimulation with LPS, leading to increases in microglia density (Furube et al., 2015), whereas those in other brain regions did not undergo microglial mitosis with such a weak inflammatory stimulation (Shankaran et al., 2007; Chen et al., 2012). Activated microglia have been shown to mediate the clearance of pathogens, cytokines, and toxic factors as well as apoptotic cells (Gordon, 2003). Therefore, microglia may play a role in the rapid clearance of toxic substances in order to protect neurons in the sensory CVOs.

## Direct sensing of blood-derived information

**Figure 5:**
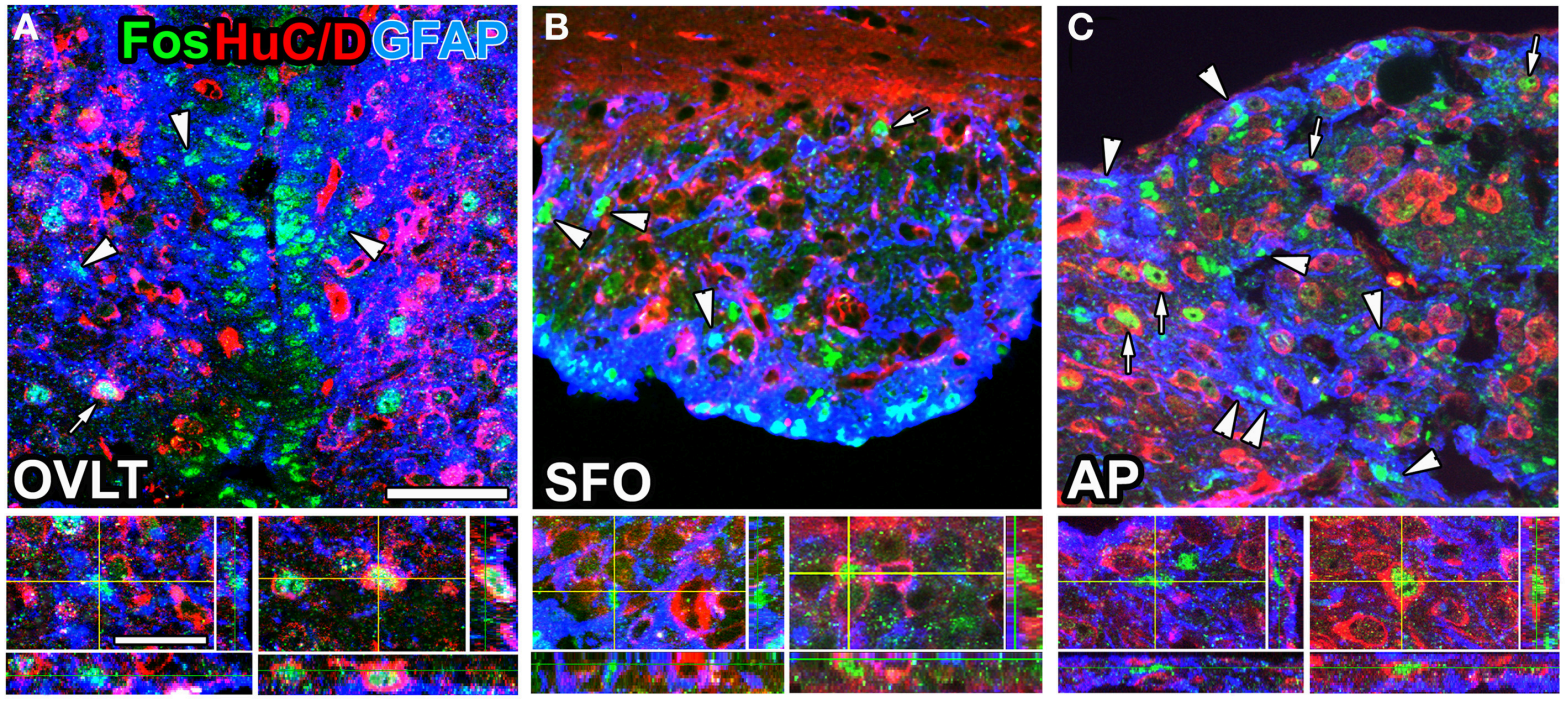
**Brain infusion of the TRPV1 agonist resiniferatoxin induced Fos expression by GFAP-positive NSCs and neurons in the sensory CVOs of adult mice**. A large number of Fos-positive nuclei were observed in the OVLT **(A)**, SFO **(B)**, and AP **(C)** after the intracerebroventricular infusion of resiniferatoxin. Fos-positive nuclei were detected in GFAP-positive NSCs (arrowheads) and HuC/D-positive mature neurons (arrows). 3D images confirmed the presence of Fos-positive nuclei in HuC/D-positive neurons and GFAP-positive NSCs. Scale bars = 10 (bottom panels) and 50 (top panels) μm. Photographs are rearranged with permission from John Wiley and Sons Inc. (Mannari et al., 2013).

The sensory CVOs directly sense blood- and CSF-derived information via sensor proteins on their parenchyma cells; however, the mechanisms by which they detect this information without causing neuronal damage currently remain unclear. The sensory CVOs are crucially involved in body fluid homeostasis. Acute and chronic hyperosmotic stimuli have been shown to induce the expression of the neuronal activity marker Fos in the sensory CVOs, while lesions in the OVLT or SFO resulted in abnormal osmotic homeostasis (Hochstenbach and Ciriello, 1996; Miyata et al., 1996; McKinley et al., 2004). Transient receptor potential vanilloid 1 (TRPV1) is a non-selective cation channel gated by mechanical/osmotic stimuli, temperature, and capsaicin (Tominaga and Tominaga, 2005). In *Trpv1*-deficient mice, the osmosensory signal transduction cascade was found to be absent in OVLT neurons, and water intake was reduced in response to a systemic hyperosmotic stimulation (Ciura and Bourque, 2006; Ciura et al., 2011). TRPV1 was recently shown to be expressed by astrocyte-/tanycyte-like NSCs in the sensory CVOs of adult mice (Mannari et al., 2013; Sladek and Johnson, 2013). Pharmacological experiments further revealed that the TRPV1 agonist resiniferatoxin preferentially induced the expression of Fos in astrocyte-/tanycyte-like NSCs rather than in neurons (Mannari et al., 2013; Figure 5). A previous study showed that hyponatremia led to the influx of water into the brain and increases in astrocyte volumes in order to preserve neuronal volumes (Ayus et al., 2008). This finding indicates that alterations in cell volume are larger in astrocytes than in neurons in order to avoid neural damage. Another important sensing protein for body fluid homeostasis is the Na^+^-sensitive Na^+^ channel Na_X_ (Hiyama et al., 2002; Watanabe et al., 2006). Na_X_ is strongly expressed by the fine cellular processes of astrocyte-/tanycyte-like NSCs in the sensory CVOs and senses angiotensin II and Na^+^ levels in the CSF (for a review, see Noda, 2006, 2007; Noda and Hiyama, 2015). Elevated levels of angiotensin II and Na^+^ in the CSF were found to trigger responses by SFO neurons (Fitzsimons, 1975; Tiruneh et al., 2013). The activation of excitatory neurons in the SFO has been shown to induce drinking behavior, even in fully water-satiated mice, whereas the activation of inhibitory GABAergic neurons markedly suppressed drinking behavior, even in thirsty animals (Oka et al., 2015).

**Figure 6:**
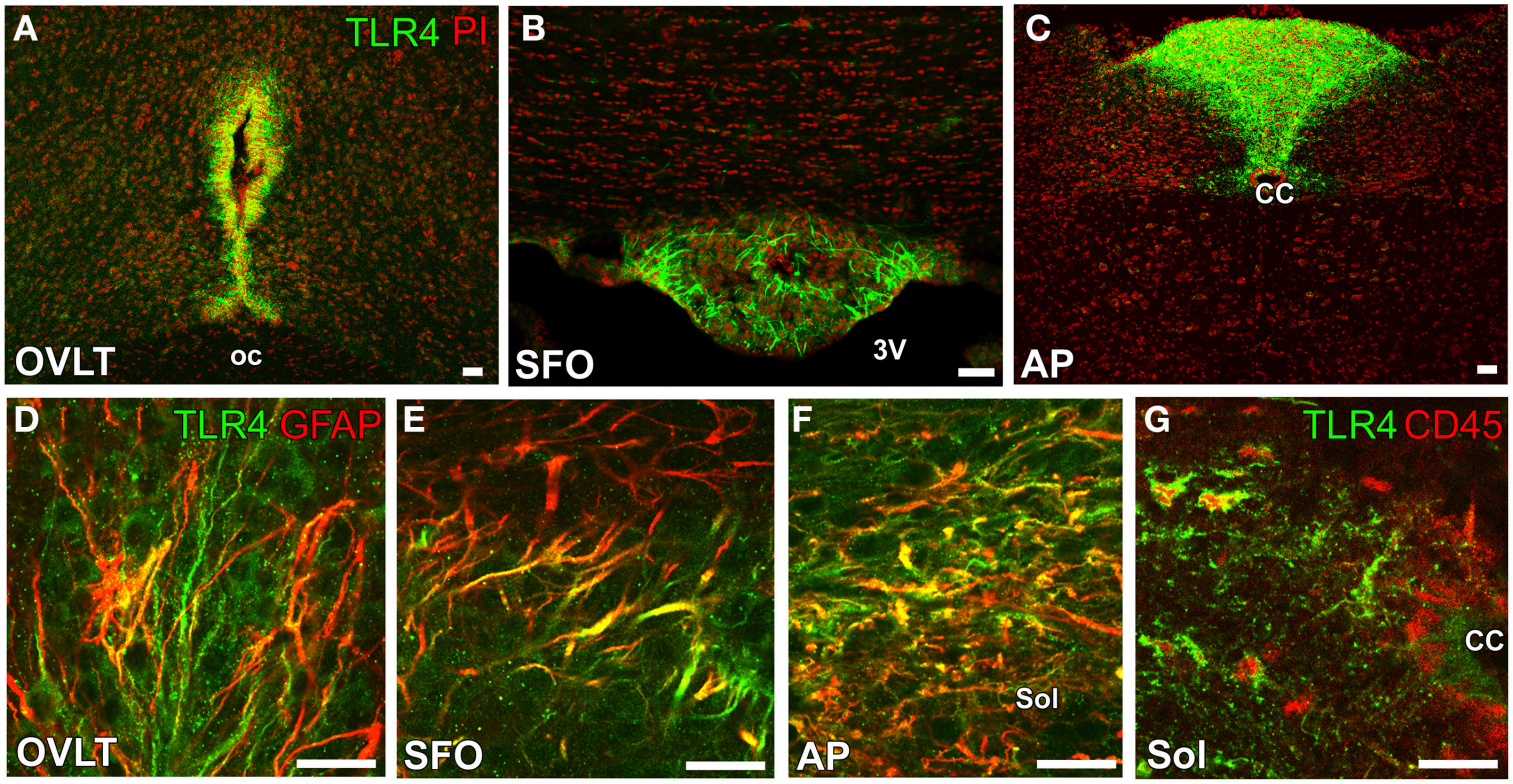
**Expression of the LPS receptor TLR4 by GFAP-positive NSCs in the sensory CVOs of adult mice**. TLR4 was strongly expressed in the OVLT **(A)**, SFO **(B)**, and AP **(C)**. The expression of TLR4 was detected in GFAP-positive NSCs in the sensory CVOs **(D–F)**. The expression of TLR4 was also observed in CD45-positive microglia in the solitary nucleus around the central canal **(G)**. CC, central canal; oc, optic chiasma; 3V, 3rd ventricle. Scale bars = 50 μm. Photographs are reconstructed with permission from Elsevier Inc. (Nakano et al., 2015).

Another, important function of the sensory CVOs is the recognition of bacteria and virus components as well as the integration of brain inflammatory responses. LPS, a component of Gram-negative bacterial walls, is a well-characterized inflammatory stimulation. IL-6 has been identified as the most abundant cytokine in the blood circulation of animals and humans after an LPS-induced inflammatory stimulation (LeMay et al., 1990), and, thus, LPS-induced inflammatory and fever responses are weaker in IL-6-deficient mice (Chai et al., 1996). IL-6 has been shown to activate the prostaglandin-synthesizing enzyme cyclooxygenase-2 in the brain, most likely via the Janus kinase and STAT3 signaling system (Akira, 1997; Rummel et al., 2005; Damm et al., 2011). Furthermore, previous studies demonstrated that the peripheral administration of LPS activated STAT3 in astrocyte-/tanycyte-like NSCs in the sensory CVOs (Gautron et al., 2002; Harré et al., 2002, 2003; Rummel et al., 2005; Nakano et al., 2015). Blood levels of LPS were also reported to be higher after the intraperitoneal administration of LPS (Lenczowski et al., 1997). Reciprocal bone marrow chimeras between wild-type and TLR4 mutant mice revealed that brain TLR4 was critically important for sustained inflammation following the peripheral administration of LPS (Chakravarty and Herkenham, 2005). *Tlr4* mRNA was previously reported to be strongly expressed in the sensory CVOs of mouse brains (Laflamme and Rivest, 2001; Chakravarty and Herkenham, 2005). A recent study showed that TLR4 was expressed by astrocyte-/tanycyte-like NSCs in the sensory CVOs (Figures 6A–F), whereas its microglial expression was restricted to a part of the solitary nucleus (Figure 6G) surrounding the central canal (Nakano et al., 2015). In addition to a peripheral LPS stimulation, the brain infusion of LPS was found to activate STAT3 signaling in the astrocyte-/tanycyte-like NSCs of the sensory CVOs (Nakano et al., 2015). NSCs of adult dorsal root ganglia have been shown to achieve longevity, multipotency, and the high fidelity of the sensory features through the expression of TRPV1 (Singh et al., 2009). Thus, astrocyte-/tanycyte-like NSCs in the sensory CVOs are multipotent NSCs that function as sensors to detect blood- and CSF-derived information via Na_X_, TRPV1, and TLR4, and also act as a diffusion barrier against blood- and CSF-derived substances and the new generation of neurons and glial cells.

It currently remains unclear how information in body fluids and inflammatory signals detected by astrocyte-/tanycyte-like NSCs is transmitted to neurons. A recent study identified lactate derived from astrocyte-/tanycyte-like NSCs as a crucial mediator in the regulation of neuronal activities in the SFO for Na^+^-intake behavior (Shimizu et al., 2007). In the hippocampal dentate gyrus, elevations in Ca^2+^ in local astrocytic processes have been shown to participate in the local tuning of transmitter release at excitatory synapses (Di Castro et al., 2011). Astrocytes are known to release various gliotransmitters, such as glutamate and ATP, in response to stimuli that increase intracellular Ca^2+^ levels (Montana et al., 2006). In the sensory CVOs, astrocyte-/tanycyte-like NSCs may express S100 in order to control Ca^2+^ homeostasis during signaling cascades (Furube et al., 2015); however, S100 is expressed in mature astrocytes, but not in NSCs in the subventricular zone (SVZ) or subgranular zone (SGZ) (Donato et al., 2013). Astrocytes cultured from S100-deficient mice were previously shown to exhibit enhanced Ca^2+^ transients in response to treatments with KCl or caffeine, suggesting that S100 plays a role in the maintenance of Ca^2+^ homeostasis in astrocytes (Xiong et al., 2000). Thus, astrocyte-/tanycyte-like NSCs directly sense blood- and CSF-derived information and then secrete certain kinds of gliotransmitters in order to activate adjacent neurons in the sensory CVOs, which may be reasonable for sensing blood- and CSF-derived information without neuronal damage. However, neurons themselves may directly receive blood- and CSF-derived information. For example, angiotensin II AT_1_ receptors were previously shown to localize in neurons and, thus, peripheral transmitters may gain direct access to neurons (Simpson et al., 1978; Frederick et al., 1984; Lippoldt et al., 1993; McKinley et al., 2003; Premer et al., 2013). Neuronal somata are typically located far from capillaries, while capillaries are surrounded by numerous layers of the cellular processes of dendrites and astrocyte-/tanycyte-like NSCs, and dendrites and axons sometimes exist within the perivascular space (Delmann, 1987; Dellmann, 1998; Morita et al., 2015a; Figure 3), suggesting that dendrites directly receive blood-derived information.

## Angiogenesis

**Figure 7:**
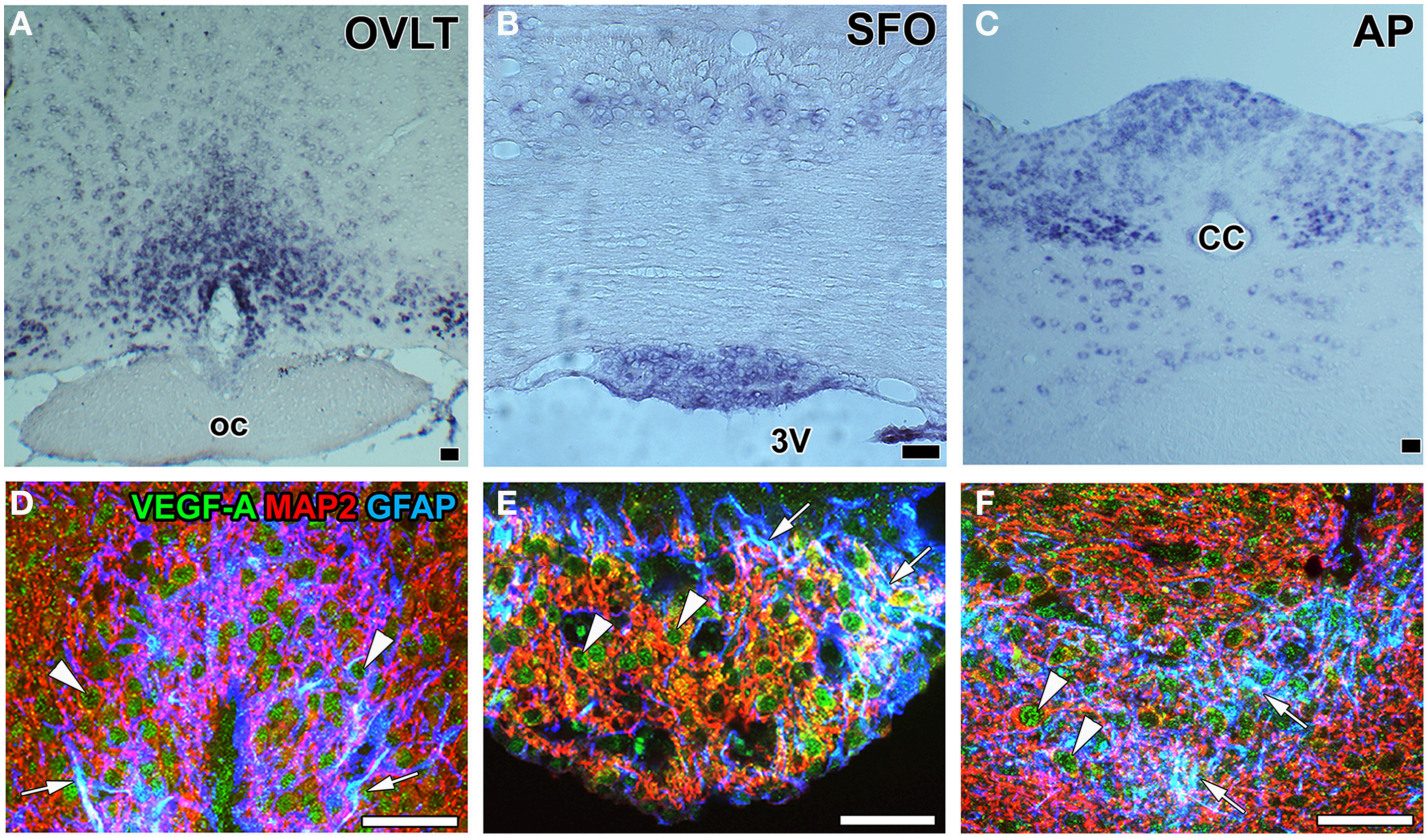
**mRNA and protein expression of the angiogenesis-inducing factor VEGF-A in the sensory CVOs of adult mouse brains**. *In situ* hybridization histochemistry shows stronger *Vegf-a* mRNA signals in the OVLT, MPA, SFO, AP, and solitary nucleus than in the adjacent brain regions **(A–C)**. Triple labeling immunohistochemistry shows that the immunoreactivity of VEGF-A was detected in GFAP-positive NSCs (arrows) and MAP2-positive mature neurons (arrowheads) **(D–F)**. CC, central canal; oc, optic chiasma; 3V, 3rd ventricle. Scale bars = 50 μm. Photographs are rearranged with permission from Springer-Verlag (Furube et al., 2015; Morita et al., 2015b).

**Figure 8:**
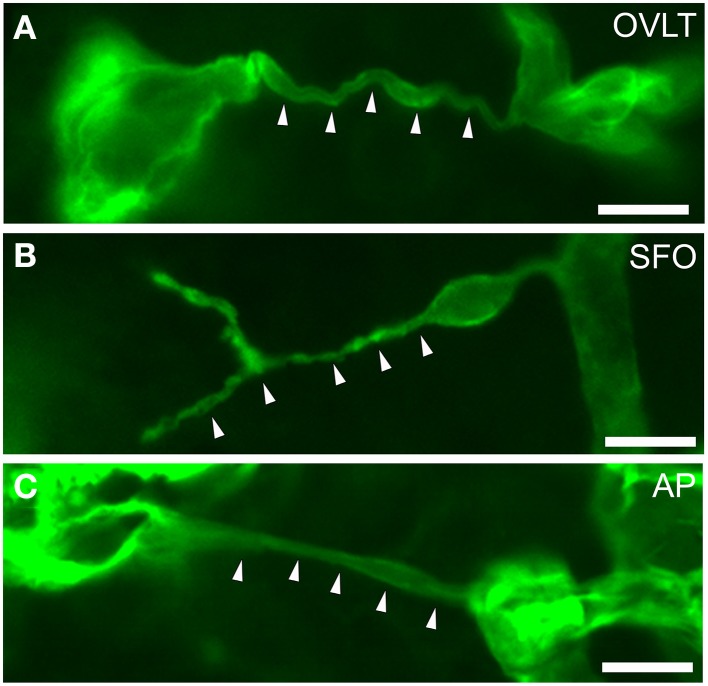
**Filopodia of endothelial cells in the sensory CVOs of the adult mouse**. Laminin-positive vascular filopodia (arrowheads) extended from the existing thick capillariesin the OVLT **(A)**, SFO **(B)**, and AP **(C)**. Scale bars = 10 μm. Confocal micrographs are rearranged with permission from Springer-Verlag (Morita et al., 2015b).

During vascular development, the process of angiogenesis and the proliferation of endothelial stalk cells and sprouting of endothelial tip cells were shown to be regulated by the concentration and gradient of VEGF-A, respectively (Gerhardt et al., 2003). The proliferation of endothelial stalk cells is known to peak 7 days after birth in the cerebral cortex (Robertson et al., 1985; Ogunshola et al., 2000; Mancuso et al., 2008), but is almost absent in the adult mammalian brain, except under pathological conditions such as injury or hypoxia (Hjelmeland et al., 2011). However, continuous angiogenesis occurs in the sensory and secretory CVOs (Morita et al., 2013b, 2015b; Furube et al., 2014). The proliferation of endothelial cells has been reported in the ME (Morita et al., 2013b) and NH (Furube et al., 2014) as well as in the OVLT, SFO, and AP (Morita et al., 2015b) of adult mice. *In situ* hybridization histochemistry revealed that VEGF-A mRNA expression levels were higher in the sensory CVOs than in adjacent brains regions (Morita et al., 2015b; Figures 7A–C). Immunohistochemistry showed that VEGF-A was highly expressed by neurons and astrocyte-/tanycyte-like NSCs in the sensory CVOs (Furube et al., 2015; Figures 7D–F) and by neuronal somata and terminals in the ME (Morita et al., 2013b). Previous studies demonstrated that the inhibition of VEGF signaling significantly attenuated the proliferation of endothelial stalk cells in the sensory and secretory CVOs (Morita et al., 2013a, 2015b; Furube et al., 2014). The sprouting of endothelial cells has been detected in the sensory CVOs of adult mouse brains (Morita et al., 2015b; Figure 8). These findings indicate that continuous angiogenesis occurs in the secretory and sensory CVOs of adult rodent brains.

**Figure 9:**
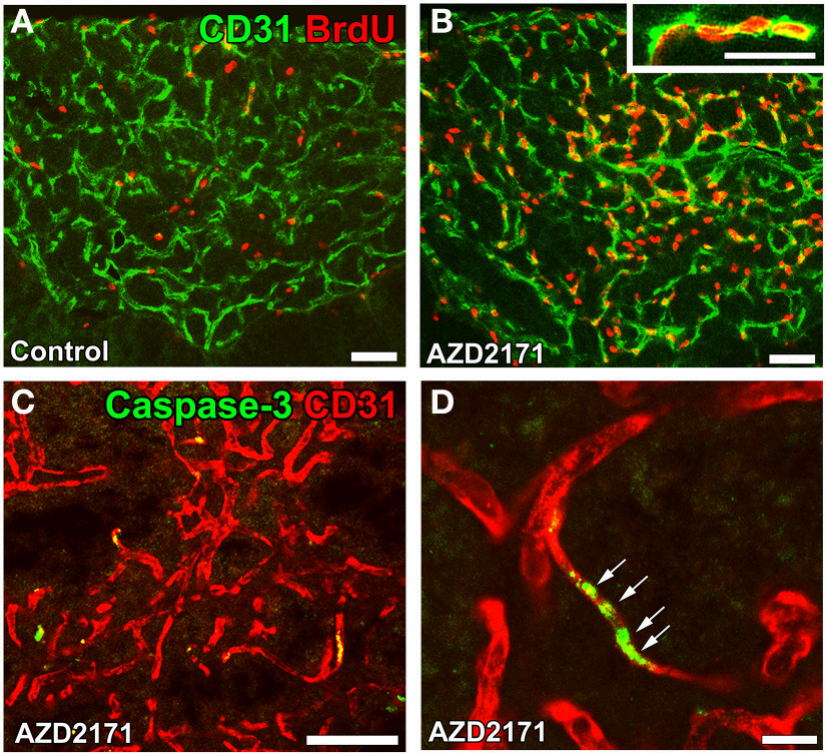
**Proliferation and apoptosis of endothelial cells in the NH of adult mice**. Mice were orally administered the VEGF signaling inhibitor AZD2171 for 6 days and were then kept for 6 days. The number of BrdU-labeled endothelial cells was significantly higher after the withdrawal of the VEGF signaling inhibitor **(B)** than that of the control **(A)**. The expression of the apoptotic marker caspase-3 was induced in endothelial cells after a 2-day treatment with the VEGF signaling inhibitor **(C)**. A high magnification view reveals the continuous distribution of caspase-3-positive endothelial cells **(D)**. Scale bars = 50 **(A–D)** and 5 (inset in **B**) μm. Data are rearranged with permission from BioScientifica Limited (Furube et al., 2014).

The functional significance of angiogenesis in the CVOs of adult brains currently remains unclear. It may play a role in long-term vascular plasticity in order to control neurosecretion and sense blood-derived information. A treatment with the VEGFR signaling inhibitor AZD2171 was found to markedly reduce vascular density by inhibiting endothelial cell proliferation (Figures 9A,B) and promoting apoptosis (Figures 9C,D) in the NH (Furube et al., 2014). Moreover, the inhibition of VEGF signaling largely decreased the density of AVP- and OXT-containing axonal terminals. The vascular surface area and its contact with the axonal terminals of AVP- and OXT-containing neurons are known to be important for efficient neurosecretion (Miyata et al., 2001; Miyata and Hatton, 2002; Imamura et al., 2010). Thus, the angiogenesis-dependent regulation of vascular density may be involved in neurosecretory and sensing activities in the CVOs.

The second possibility is that VEGF-dependent angiogenic activity is associated with the states of the fenestrated features. The brain infusion of VEGF-A was previously reported to decrease the expression of the tight junction proteins claudin-5 and occludin and induced barrier breakdown in the cerebral cortex of adult mice (Argaw et al., 2009). The vascular permeability of HMW substances was found to be negligible in fetal and adult brains (Armulik et al., 2010; Daneman et al., 2010), whereas that of LMW substances was higher in the immature capillaries of fetal brains than in those of adult brains (Tuor et al., 1992; Keep et al., 1995). Thus, the size-selective permeability of fenestrated capillaries in the CVOs is similar to the angiogenic immature capillaries of fetal brains. The mitotic inhibitor cytosine-b-D-arabinofuranoside has been shown to decrease the proliferation of endothelial cells and vascular permeability to blood-derived LMW molecules without changing the vascular area or diameter (Morita et al., 2015b). A recent study demonstrated that pericytes play important roles in the formation of the BBB during embryogenesis (Daneman et al., 2010), with the loss of pericytes resulting in BBB disruption (Armulik et al., 2010). Chronic salt loading is known to increase the pericytic expression of platelet-derived growth factor receptor ßin the sensory CVOs in combination with elevations in vascular permeability (Morita et al., 2014). Furthermore, food and glucose deprivation increased the expression of PV-1 in the fenestrated capillaries of the ME, tight junction proteins in tanycytes, and vascular permeability of ME capillary loop, thereby promoting metabolic substrate access to the Arc and feeding behavior (Langlet et al., 2013a). The expression of VEGF-A mRNA has also been shown to be upregulated by food and glucose deprivation, while the inhibition of VEGF signaling abolished the food deprivation-induced reorganization of tanycytes and capillaries as well as food intake behaviors (Langlet et al., 2013a). Thus, the angiogenesis-associated factors VEGF-A and platelet-derived growth factor may largely affect the states of the fenestrated features in the CVOs.

A final possibility is that angiogenesis engages in the maintenance and proliferation of NSCs and their associated structural reconstruction. In the hippocampal dentate gyrus, VEGF-A has been shown to regulate the proliferation of endothelial cells and NSCs in a coordinated manner (Warner-Schmidt and Duman, 2007; Segi-Nishida et al., 2008; Udo et al., 2008). A previous study reported that the overexpression of VEGF-A significantly increased angiogenesis and neurogenesis in the adult hippocampus (Udo et al., 2008). Electroconvulsive seizures and antidepressants, which are proven therapeutics in the treatment of several depressive diseases, increased vascular density in the DG of adult rodents and humans (Newton et al., 2006; Mannari et al., 2014). NSCs have a perivascular niche that intimately associates with endothelial cells, possibly via VEGF and BDNF signaling, in the SGZ and SVZ of adult mammalian brains (Goldman and Chen, 2011). A causal interaction has been reported between testosterone-induced angiogenesis and neurogenesis in adult songbird canary brains (Louissaint et al., 2002). NSCs have also been detected in the sensory CVOs of mice and humans (Bennett et al., 2009; Sanin et al., 2013; Furube et al., 2015). Moreover, neural progenitor cells (NPCs) have been associated with the vascular matrix (Hourai and Miyata, 2013). Thus, tissue dynamics including neurogenesis and gliogenesis are regulated with angiogenesis in a coordinated manner.

## Neural stem cells

The generation of new neurons and glial cells continuously occurs at restricted brain regions in adult mammals. The most extensively examined brain regions are the SGZ, located in the dentate gyrus of the hippocampus (Eriksson et al., 1998; Gage, 2000), and the SVZ, lining the lateral ventricle (Doetsch et al., 1999). A deficiency in neurogenesis in the SGZ was found to disrupt negative hippocampal control in the hypothalamic-pituitary-adrenal axis, thereby leading to depressive illnesses (Snyder et al., 2011), while that in the SVZ led to the lack of predator avoidance and sex-specific responses (Sakamoto et al., 2011), indicating that neurogenesis is a region-specific function.

**Figure 10:**
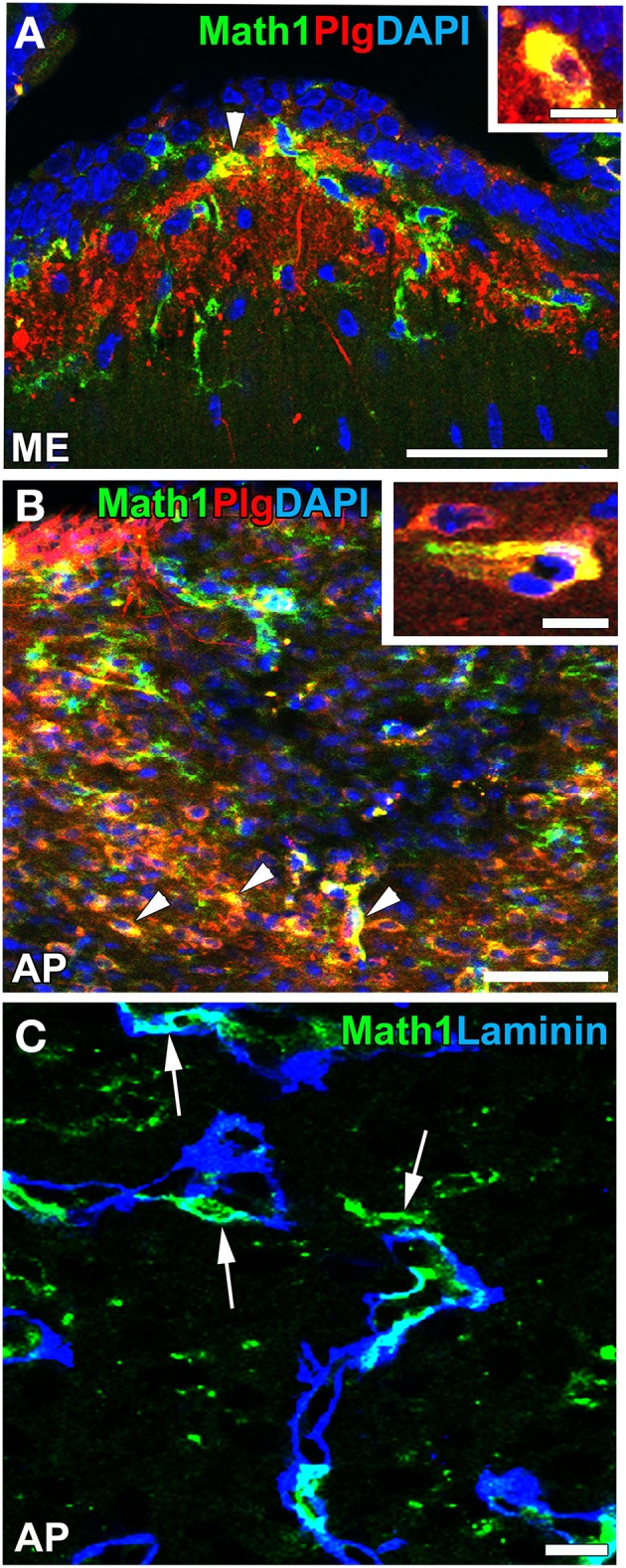
**Plasminogen expression and vascular niche of Math1-positive NPCs in the ME and AP of adult mice**. Strong immunoreactivity for plasminogen was detected in Math1-positive NPCs (arrowheads) in the ME **(A)** and AP **(B)**. Math1-positive NPCs typically localized in close contact with the vascular matrix in the AP (arrows). DAPI, diamidino-2-phenylindole; Plg, plasminogen. Scale bars = 50 **(A,B)** and 10 (**C**, insets in **A,B**) μm. Photographs are reconstructed with permission from John Wiley and Sons Inc. (Hourai and Miyata, 2013).

Recent findings indicated that NSCs are also present in the CVOs of adult mammalian brains. A neurosphere assay *in vitro* indicated that NSC-like cells were present in the ependymal layers of the third and fourth ventricles of adult mouse brains (Weiss et al., 1996). The intracerebroventricular infusion of fibroblast growth factor-2 and epidermal growth factor has been shown to induce the proliferation of NSC-like ependymal cells in the third and fourth ventricles of adult mice *in vivo* (Martens et al., 2002; Xu et al., 2005). _2_ tanycytes at the base of the third ventricle in the ME have been proposed as NSCs that proliferate and give rise to new neurons and glial cells (Lee et al., 2012). In contrast, a previous study reported that α_2_ tanycytes in the ME and Arc were able to self-renew or give rise to β_2_ tanycytes and parenchyma cells *in vivo* and exhibited stem-like neurospherogenic activity *in vitro* (Robins et al., 2013). The inhibition of neurogenesis in the ventrobasal hypothalamus by focal irradiation led to weight gain in high fat diet-fed mice (Lee et al., 2012). Ciliary neurotrophic factor was found to enhance neurogenesis in the ventrobasal hypothalamus and induce weight loss in adult mice, while the mitotic inhibitor cytosine-b-D-arabinofuranoside eliminated the proliferation of neural cells and abrogated the long-term effects of ciliary neurotrophic factor on body weight (Kokoeva et al., 2005). A previous study showed that neurogenesis was attenuated in the Arc of obese mice induced by the consumption of a high-fat diet or leptin deficiency (McNay et al., 2014). Furthermore, continuous neurogenesis was detected in the ME and Arc of adult human brains (Sanin et al., 2013; Batailler et al., 2014). NPCs in the adult mouse ME have been shown to express high levels of plasminogen (Taniguchi et al., 2011; Hourai and Miyata, 2013; Figures 10A,B), the activation of which is crucial for the migration of granular neurons to the developing cerebellum *in vivo* (Seeds et al., 1997) and neuritogenesis *in vitro* (Farias-Eisner et al., 2001; Gutiérrez-Fernández et al., 2009). Moreover, NPCs were often localized in close proximity to the vasculature (Figure 10C). These findings support the presence of NSCs in the ME of adult mammalian brains (for a review, see Bolborea and Dale, 2013).

**Figure 11:**
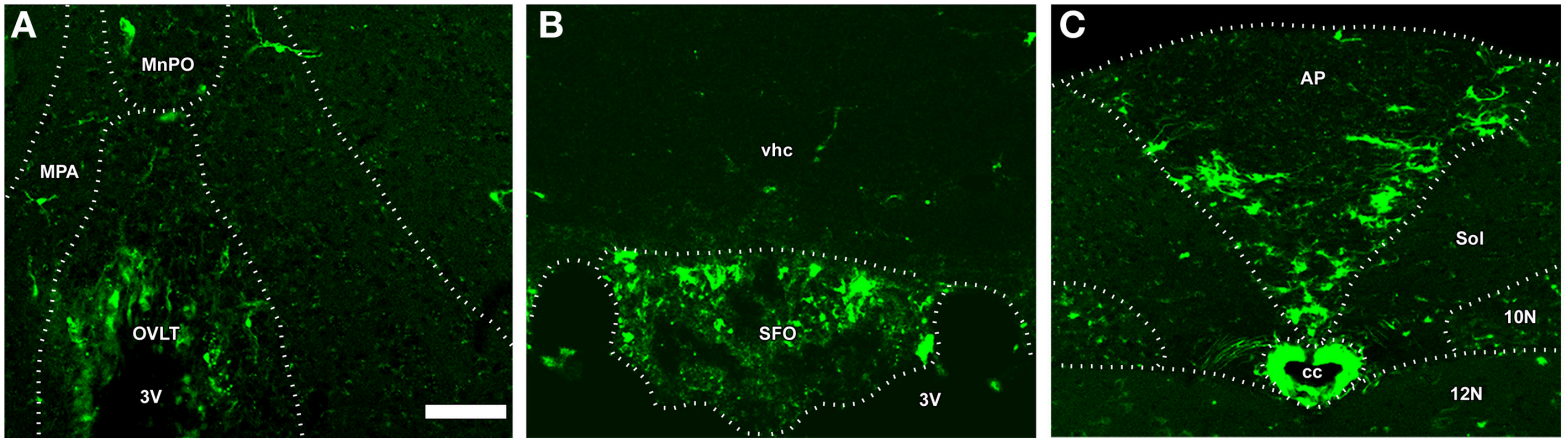
**The fate of NSCs in the sensory CVOs using ***Nestin-CreERT2/CAG-CAT***^***loxP***∕***loxP***^***-EGFP*** transgenic adult mice**. *Nestin-CreERT2/CAG-CAT*^*loxP*∕*loxP*^*-EGFP* transgenic mice were sacrificed 60 days after the final administration of tamoxifen. A large number of EGFP-expressing cells were detected in the OVLT, whereas only a few were observed in the median preoptic area and medial preoptic nucleus **(A)**. EGFP-expressing cells were observed in the vhc as well as in the SFO **(B)**. EGFP-expressing cells were detected in the AP and its neighboring brain regions such as the solitary nucleus, 10N, and 12 N **(C)**. MnPO, median preoptic area; MPA, medial preoptic area; Sol, solitary nucleus; vhc, ventral hippocampal commissure. Scale bar = 50 μm. Data are rearranged with permission from Springer-Verlag (Furube et al., 2015).

In addition to the ME, NSCs were recently detected in the sensory CVOs such as the OVLT, SFO, and AP (Bennett et al., 2009; Hourai and Miyata, 2013; Furube et al., 2015). The presence of NCSs in the sensory CVOs was confirmed by a neurosphere experiment *in vitro* (Bennett et al., 2009). Two types of NSCs may exist in the sensory CVOs: tanycyte-like NSCs located at the ependymal layer and astrocyte-like NSCs at the parenchyma (Furube et al., 2015; Figure 11). Tanycyte-like ependymal cells in the sensory CVOs are devoid of cilia, have long cellular processes, and closely resemble those in the ME (Rodríguez et al., 2005; Mullier et al., 2010; Langlet et al., 2013a). However, the characterization of tanycytes, especially a subtype analysis in the sensory CVOs, has not yet been conducted, unlike the ME (Rodríguez et al., 2005). NSCs have also been detected in the AP of adult human brains (Sanin et al., 2013). Although astrocyte-like NSCs proliferate slowly, oligodendrocyte progenitor cells (OPCs) and NPCs actively divide (Furube et al., 2015). The inhibition of VEGF signaling and peripheral administration of LPS were found to significantly suppress the proliferation of NSCs and OPCs (Furube et al., 2015). These findings indicate that NSCs are present in the sensory CVOs, such as the OVLT, SFO, and AP, of adult mammalian brains. However, further cell lineage analyses of NSCs are needed in order to determine neurogliogenesis in the sensory CVOs, differences between tanycyte- and astrocyte-like NSCs, and characterize the tanycyte subtypes possessing neurogenic activity.

A fate mapping study reported that NSCs mainly gave rise to oligodendrocytes and a sparse number of neurons and astrocytes (Furube et al., 2015). NSCs originating from the OVLT may migrate into adjacent hypothalamic brain regions such as the medial preoptic area and median preoptic nucleus, while those derived from the AP may migrate into the solitary nucleus (Furube et al., 2015). Thus, the sensory CVOs may supply new cells to the adjacent hypothalamic and medullar regions and also to the sensory CVOs themselves. The proliferation of NSCs and their progenitor cells was previously shown to be facilitated by ischemic stroke injury (Lin et al., 2015). However, although the functional significance of NSCs in the sensory CVOs currently remains unknown, they have been assumed to participate in the long-term control of the sensory CVO functions, such as body fluid homeostasis and neuroinflammation.

### Conflict of interest statement

The author declares that the research was conducted in the absence of any commercial or financial relationships that could be construed as a potential conflict of interest.
